# Diagnostic Dilemma: Primary Peritoneal Mesothelioma With Para-Occupational Asbestos Exposure

**DOI:** 10.1200/JGO.2016.005280

**Published:** 2016-08-31

**Authors:** Kirby R. Qin, Divyanshu Dua

**Affiliations:** **Kirby R. Qin**, Monash University; **Divyanshu Dua**, Mildura Base Hospital, Victoria, Australia.

## INTRODUCTION

Mesotheliomas are rare neoplasms arising from mesothelial cells that cover the peritoneum, pleura, pericardium, and tunica vaginalis.^1^ We present the case of a 62-year-old woman with primary malignant peritoneal mesothelioma (MPM).

## CASE REPORT

A 62-year-old woman presented to a hospital in rural Australia with complaints of left lower quadrant abdominal pain, bloating, dyspnea, and a dry cough. She reported weight loss of 4 kg over 2 months (previously, 48.5 kg), fevers, and fatigue. Her medical history was significant for chronic constipation since age 20 years, and hemorrhoidectomy at age 59 years. Physical examination revealed moderate abdominal distension with mild tenderness. The patient recalled domestic exposure to asbestos from her husband, who was an insulation worker.

**Fig 1:**
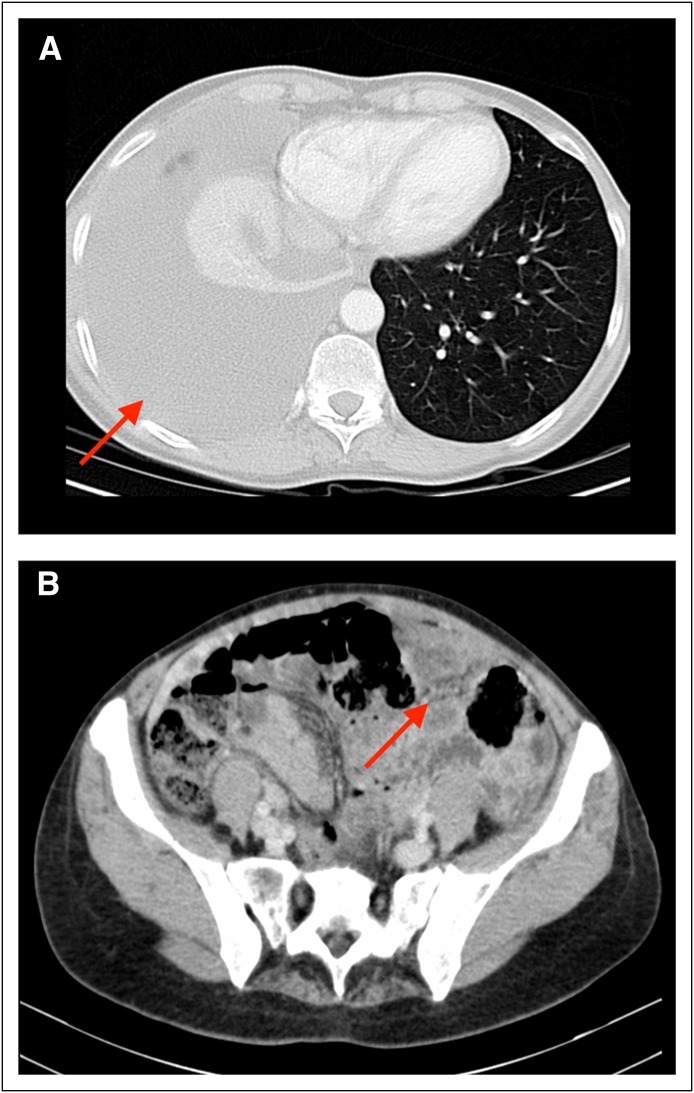
Computed tomography images of the chest and abdomen. (A) Right-sided pleural effusion with right lower lobe atelectasis (arrow). (B) Omental thickening in the left flank (arrow).

Biochemical studies revealed deranged liver function test results, including albumin level of 30 g/L, alkaline phosphatase, 129 U/L; gamma-glutamyl transferase, 66 U/L; and ALT, 53 U/L. A computed tomography (CT) scan of the chest displayed atelectasis of the right lower lobe and right-sided pleural effusion. An abdominal CT scan revealed thickening of the omentum with fine nodularity in the left flank. Importantly, no ascites, lymphadenopathy, or ovarian pathology could be appreciated (Fig 1).

Within the course of 6 weeks, thoracocentesis was performed on four separate occasions to manage pleural effusion. Each tap returned more than 1 L of pleural fluid. Atypical mesothelial cells were found in the pleural aspirate; malignant cells were absent. Surgical pleurodesis was performed to limit future effusion.

Laparoscopic exploration of intra-abdominal contents was conducted because pleural fluid and CT scan had failed to establish a diagnosis. Gross appearance of the peritoneum revealed numerous peritoneal nodules in the left iliac fossa, several umbilical nodules, and apparent umbilical thickening. A small trace of peritoneal fluid was found in the pelvic region. The sigmoid colon was adhered to the anterior abdominal wall via filmy adhesions. The adhesions were surgically divided, and samples were collected for testing. Biopsy specimens were collected from 12 tan peritoneal nodules and ranged from 2 to 9 mm in diameter. A 12 mm by 9 mm section of variegated umbilical tissue was also collected. Both samples contained cells exhibiting neoplastic changes, and both immunostained positive for MPM markers. Peritoneal fluid showed clumps of malignant cells. After diagnosis, chemotherapy was commenced.

## DISCUSSION

The peritoneal cavity is the second most common site for mesothelioma. In the United States, of 10,589 mesothelioma cases reported between 1973 and 2005, 10.5% were peritoneal.^2^ Incidence among industrialized nations ranges between 0.5 and 3 cases per million in men, and between 0.2 and 2 cases per million in women.^3^

There is a strong relationship between asbestos exposure and mesothelioma. The lifetime risk of developing mesothelioma in asbestos workers is thought to be as high as 10%, with a mean latency period of 30 years.^4^ Furthermore, approximately 80% of patients with mesothelioma report a history of asbestos exposure.^5^ The association between asbestos and peritoneal mesothelioma is less than that of pleural mesothelioma. This may be due, in part, to asbestos-induced MPM generally requiring a higher cumulative dose.^6^ Mesothelioma can also result from nonoccupational or para-occupational exposure to asbestos. For example, women whose husbands work in asbestos-related industries often come in contact with asbestos while laundering their husband’s work-related clothing^7^; this is the likely cause in this report.

Excluding asbestos, abdominal radiotherapy is the only well-documented cause of mesothelioma.^8^ Genetic predisposition is related to mutations of the *BAP1* gene^9^; however, the overall magnitude of risk remains undefined.

Clinical presentation of MPM is generally nonspecific. Patients complain of abdominal distension, pain, nausea, anorexia, and weight loss. Complications of bowel obstruction, such as constipation and vomiting, tend to manifest with advancing disease. It is unclear whether our patient’s constipation was an independent symptom or a result of peritoneal disease. Left lower quadrant tenderness may be explained by the discovery of extensive nodular formations. Pleural spread of malignant disease was likely the causative factor behind this patient’s respiratory symptoms.

MPM has been studied to a lesser extent than its pleural counterpart. Thus, there is no definitive guideline detailing the most effective diagnostic strategy. Radiographic findings are especially uncertain, and are often inconsistent between patients.

CT is an essential study in any patient with abdominal pain and distension. In some reported cases, MPM manifests as diffuse peritoneal disease with malignant infiltration and nodular thickening of the parietal peritoneum in a sheet-like fashion.^10^ As the tumor progresses, growth may extend to the visceral peritoneum of the bowels, resulting in the formation of abdominal adhesions. MPM also exhibits involvement of the omentum, diaphragm, liver, small and large bowels, and mesentery. Implication of the pleura is also a frequent finding in patients with MPM, specifically pleural calcification and recurrent pleural effusions.^11^ For our patient, CT findings were nonspecific, warranting further investigation.

Although CT is the gold-standard imaging modality in mesothelioma, research suggests imaging features are comparable with magnetic resonance imaging (MRI). MRI has been shown to more accurately predict burden of disease in posttreatment follow-up.^12^ Integrated positron emission tomography (PET)-CT has also been implemented in assessing pleural mesothelioma; however, it is unclear whether results are replicable in the peritoneum.^13^ Neither MRI nor PET are considered standard practice in Australia.

Because of inconclusive radiographic evidence, direct visualization of intra-abdominal contents via laparoscopy was necessary to determine the cause of disease in this case. Both laparoscopy and laparotomy are associated with high diagnostic performance in the investigation of MPM.^14^ However, minimally invasive laparoscopic exploration is associated with improved patient outcomes.

The macroscopic appearance of MPM is typically characterized by the presence of white tumor nodules on the parietal peritoneum.^15^ There are usually hundreds to thousands of nodules, which vary in size and consistency. Histologic examination of peritoneal nodules differentiates MPM into three subtypes: epithelioid, sarcomatoid, and biphasic.^16^ The epithelioid variant is most common, constituting up to 75% of cases. Epithelioid mesotheliomas are composed of flattened or cuboidal cells that closely resemble normal mesothelial cells. Mitotic figures are uncommon. Architecturally, they form a tubulopapillary or trabecular pattern and are predominantly composed of acinar structures.^17^ The morphology of epithelioid-type cells closely resembles that of an adenocarcinoma; hence, the presence of metastatic adenocarcinoma should be considered.

In the present case, numerous peritoneal nodules were discovered throughout the left iliac fossa. These nodules displayed an outer layer of flattened mesothelial cells. Tissue fragments showed small acinar-like structures and irregular strands of cells showing uniform nuclei. Mitotic figures were inconspicuous, with cells displaying occasional prominent nucleoli. The stroma was mildly myxoid with a few hemosiderin-laden macrophages. Umbilical tissue displayed similar flattened cells with nuclear enlargement, moderate pleomorphism, large nucleoli, and surrounding desmoplasia. These findings were consistent with epithelioid-type MPM.

**Fig 2:**
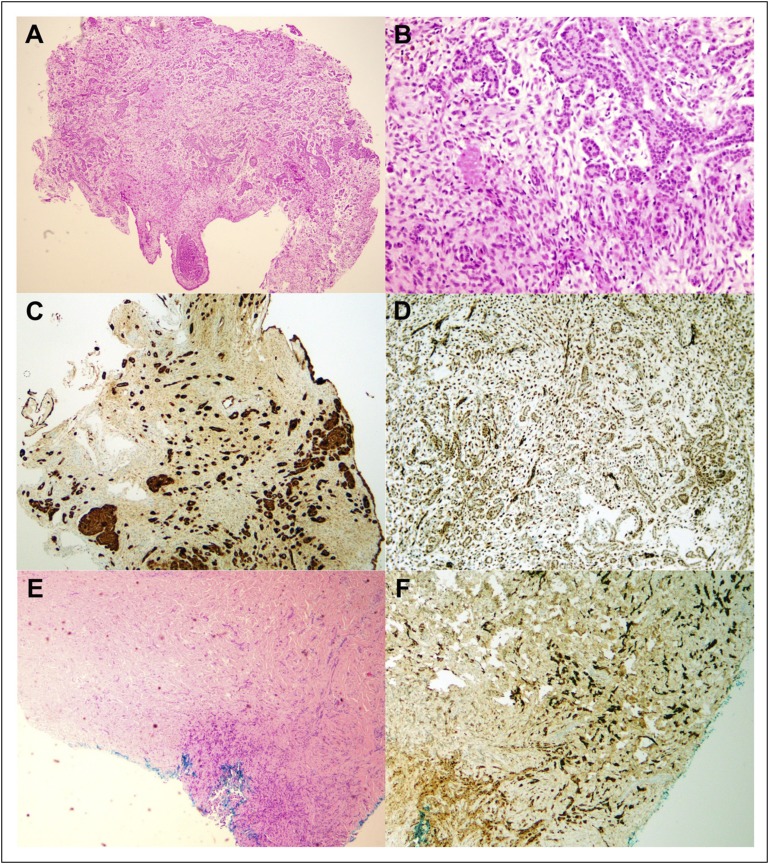
(A-D) Micrographs of peritoneal nodules. (A) Hematoxylin and eosin (HE) stain (magnification, ×10). (B) HE stain (magnification, ×40). (C) Positive stain for calretinin (magnification, ×10). (D) Wilms tumor 1 antigen (magnification, ×40). (E-F) Umbilical mass. (E) HE stain (magnification, ×10). (F) Positive stain for calretinin (magnification, ×10).

Immunohistochemistry is required in combination with macroscopic analysis to differentiate MPM from neoplastic mimics, such as metastatic adenocarcinoma. There is no single marker with sufficiently high sensitivity and specificity for MPM. Therefore, standard practice dictates the need for a panel of markers, both positive and negative. The International Mesothelioma Interest Group recommends that markers considered in diagnosis should have either sensitivity or specificity greater than 80%.^18^ Staining for pancytokeratins is especially useful because most epithelioid and sarcomatoid mesotheliomas will be positive. The suggested panel consists of a pancytokeratin in conjunction with at least two mesothelioma and two carcinoma markers. For epithelioid mesothelioma, the most common positive markers are calretinin, cytokeratinin 5/6 (CK5/6), Wilms tumor-1 (WT1) antigen, and podoplanin.^18^ All biopsy specimens obtained from our patient were positive for calretinin, CK5/6, and WT1 (Fig 2), and negative for PAX8, mammoglobin, CK20, CDX2, and GCDFP15l.

Recent studies have shown that negative staining for *BAP1* can be used to support a pathologic diagnosis of abdominal mesothelioma over serous carcinoma.^19^ However, such findings were published after our diagnosis had been made. Furthermore, fluorescence in situ hybridization analysis of *p16* gene deletion could have been used to expedite the diagnostic process.^18^

Mesotheliomas often present with serous effusions because of fluid produced by malignant cells. Fluid testing is generally unreliable in the diagnosis of malignant mesothelioma. Sensitivity reported in previous research ranged from 32% to 76%.^18^ The significant morphologic overlap between nonmalignant reactive mesothelial cells and malignant mesothelial cells may be partly responsible for this high false-negative rate. Both cell types tend to exhibit cell clumps, intercellular windows, lighter dense cytoplasm edges, and low nuclear-cytoplasmic ratios. Fluid harvested in epithelioid mesotheliomas display large clumps of malignant cells, with most much larger than normal mesothelial cells.

Mortality from MPM is generally based on disease progression within the peritoneal cavity. During later stages of disease, MPM may extend into the pleural cavity, resulting in pleural effusion.^11^ In patients presenting with pleural effusion, clinicians should investigate whether the primary is located in the pleural or peritoneal cavity. Secondary pleural effusion is typically free of malignant cells.^18^

Contrary to expected findings, our patient presented with recurrent pleural effusion and minimal peritoneal effusion. Pleural fluid analysis reported atypical mesothelial cells on a background of erythrocytes and mononuclear cells. These atypical cells exhibited reactive morphology, hyperchromatic nuclei, and enlarged nucleoli. Immunostaining revealed no evidence of malignancy. Peritoneal fluid obtained during exploratory laparoscopy demonstrated clumps of malignant cells on a background of erythrocytes, mixed leukocytes, and reactive mesothelial cells. The malignant cells stained positive for calretinin, CK5/6, CK7, and WT1. The patient is currently alive and her condition has stabilized after four cycles of carboplatin and pemetrexed.
